# TiO_2_ Nanoparticles Obtained by Green Synthesis: Characterization and Evaluation of Their Effect on the Self-Cleaning and Antifungal Properties of an Aqueous Paint-Type Coating

**DOI:** 10.3390/nano16020091

**Published:** 2026-01-10

**Authors:** Kendell Alcazar, Laura Tous, Adriana Herrera, Dylan Martinez-Bernett, Manuel Saba

**Affiliations:** 1 Nanomaterials and Computer-Aided Process Engineering Research Group, Department of Chemical Engineering, College of Engineering, University of Cartagena, Cartagena 130015, Colombia; kalcazarg@unicartagena.edu.co (K.A.); ltousm@unicartagena.edu.co (L.T.); 2 Structures, Construction and Heritage Research Group, Department of Civil Engineering, College of Engineering, University of Cartagena, Cartagena 130015, Colombia; msaba@unicartagena.edu.co

**Keywords:** TiO_2_ nanoparticles, coating, self-cleaning, contact angle, photodegradation, antifungal efficacy

## Abstract

This work presents a green chemistry route to obtain titanium dioxide TiO_2_ nanoparticles with an average size of about 13.25 nm using lemongrass (*Cymbopogon citratus*) extract. For these assessments, TiO_2_ nanoparticles were added to the coating at concentrations of 1% and 5% *w*/*w* on fiber-cement sheets. Self-cleaning evaluation was analyzed by the photodegradation of methylene blue (MB) dye at concentrations of 5, 10, and 20 mg/L applied to the coated sheet, and then exposed to simulated sunlight. The coating containing 5 wt% TiO_2_ nanoparticles showed the highest photodegradation, reaching 93.3% after 4 h under simulated sunlight exposure at the lowest MB concentration (5 mg/L). Additionally, average contact angles of 80.4°, 92.03°, and 104.25° were determined for coatings containing 0%, 1%, and 5 wt% TiO_2_, respectively. Moreover, the modified 5 wt% TiO_2_ exhibited up to 30.9% greater hydrophobicity than the control. Antifungal efficacy against *Aspergillus niger* and *Penicillium* was evaluated using the Poisoned Food method with nanoparticles at concentrations of 1 and 3 mg/mL showing a moderate growth inhibition. In conclusion, the versatility demonstrated suggests potential applications such as a nano-additive for aqueous acrylic coatings, improving hydrophobicity, self-cleaning and antifungal properties, which could be attractive to the construction industry.

## 1. Introduction

Advances in nanotechnology have transformed material engineering, enabling the development of multifunctional surfaces with improved mechanical, chemical, and biological performance [1,2,3]. The incorporation of nanomaterials into paints, coatings, and polymer matrices enhances engineering applications, including environmental remediation, self-cleaning effect, and the creation of protective barriers to prevent the proliferation of microorganisms such as bacteria or fungi [4,5,6,7]. In this context, titanium dioxide (TiO_2_) nanoparticles have become one of the most studied nanomaterials due to their chemical stability, strong oxidative potential under irradiation, and compatibility with diverse substrates. Moreover, the capacity of these nanoparticles for the photodegradation of organic contaminants, self-cleaning applications, and the creation of protective barriers to prevent microorganism proliferation, makes TiO_2_ nanomaterials a core component in the development of innovative and protective coatings [4,6]. Man Ching Chen and collaborators [8] reported a review study focused on the usability of photocatalytic metal oxide nanomaterials (TiO_2_ and ZnO), as well as silver nanoparticles, for the preparation of functional paints and coatings, including the application of quaternary ammonium salts to improve antimicrobial behavior.

The strong photocatalytic activity of TiO_2_ nanoparticles can be related to their anatase crystalline phase, which facilitates the oxidative degradation of organic pollutants [9,10,11,12]. Salehzadeh and co-workers [13] studied the synthesis of TiO_2_ nanoparticles using the sol–gel method, obtaining nanoparticles with sizes from 25 to 50 nm. These nanomaterials were used for the photocatalytic degradation of malachite green dye under UV irradiation, achieving complete decolorization. These findings reinforce the strong dependence of photocatalytic efficiency on physicochemical properties such as crystalline, morphology, surface area, and band gap [9]. However, many of these systems rely on conventional chemical synthesis routes that can involve the use of toxic reagents, high energy consumption, and limited environmental sustainability [10,11,12].

As an alternative, green synthesis offers a promising approach to obtaining metal oxide nanoparticles by employing environmentally friendly pathways that incorporate plant extracts, which are rich in phytocomponents or biomolecules that can drive the reduction of inorganic precursors, while allowing the stabilization and size of the synthesized nanomaterials. Deliza and co-workers [14] indicated that the peel extract from *Baccaaurea racemosa* provides phenolic and flavonoid phytochemicals that can act as reducing and capping agents during TiO_2_ formation. From this work, they reported the synthesis of TiO_2_ nanoparticles with high crystallinity, a small size (32 nm), and a good colloidal stability, demonstrating effective photocatalytic performance, as evidenced by the photodegradation of 99% of the Acid Red-185 dye. These advantages underscore the potential of plant-mediated synthesis to produce functional nanomaterials with reduced environmental impact.

The use of *Cymbopogon citratus* extract has received particular attention due to its high content of citral, terpenoids, and phenolic compounds, which confer strong reducing capability during nanoparticle formation. In plant-mediated green synthesis routes, these phytochemicals are widely recognized to act as multifunctional reducing and stabilizing agents, enabling nanoparticle formation without the need for additional chemical reagents [15]. Compared to other plant extracts commonly employed in green synthesis, lemongrass extract offers practical advantages, such as simple aqueous extraction and good reproducibility, as discussed in comparative reviews of plant-based nanoparticle synthesis [16]. The study by Patiño-Ruiz and collaborators [17] demonstrated the capacity of an aqueous lemongrass extract to reduce metal ions during the formation of iron oxide nanoparticles, stabilizing nanoparticle surfaces and simultaneously improving biocompatibility. Similarly, in the context of photocatalytic and antimicrobial applications, Vijayakumar and co-workers [18] confirmed the synthesis of TiO_2_ and Ag–TiO_2_ nanoparticles in the presence of lemongrass extract, obtaining average sizes of approximately 12 nm and 42 nm, respectively. These nanomaterials exhibited effective dye degradation (up to 78% for TiO_2_ and 97% for Ag–TiO_2_ nanoparticles under sunlight) and excellent antimicrobial activity, suggesting the environmental and functional advantages of implementing plant-based synthesis to obtain photocatalytic nanomaterials.

Regarding the green synthesis of TiO_2_ nanoparticles, several factors can affect nanomaterials’ physicochemical and optical properties, including plant source, extraction method, and reaction conditions, such temperature and time [19]. A comparative study published by Sangeetha and collaborators [20] indicates that solvent selection plays a critical role in the formation of TiO_2_ nanomaterials. In this work, they prepared TiO_2_ nanoparticles using different solvent media, including isopropanol with acetic acid and natural extracts from Jasminum and Magnolia champaca flowers. They observed the formation of the anatase crystalline phase in the synthesized nanoparticles after using the chemical solvent, whereas the flower extracts yielded the rutile crystalline phase. Moreover, a broader size distribution was observed for the nanoparticles prepared with the flower extracts. These discrepancies indicate a lack of standardized synthesis parameters within green chemistry approaches, complicating efforts to optimize nanoparticles for specific coating applications.

Despite substantial progress documented in the literature, several limitations persist across the broader scientific landscape. Most research focuses on nanoparticle synthesis and basic characterization rather than integrating green-synthesized TiO_2_ into functional coating systems. Many studies assess photocatalysis or antimicrobial activity in isolation, limiting insights into the multifunctional performance in real-world settings. Additionally, substrate-specific evaluations, particularly for construction materials such as fiber cement, are scarce, even though substrate porosity, surface roughness, and environmental exposure strongly influence coating behavior. Another unaddressed gap is the optimal loading of green-synthesized TiO_2_ nanoparticles in coatings, as excessive concentrations can induce agglomeration and diminish coating uniformity.

The coatings industry faces the challenge of developing and promoting products that not only act as physical barriers but also incorporate desirable functional properties, such as antifungal and self-cleaning capabilities. This limitation stems from a lack of innovation in formulations that simultaneously combine encapsulant performance, sustainability, and multifunctionality. Currently, the domestic market lacks a paint that effectively integrates all these characteristics, representing a technical and commercial opportunity for the development of improved products which could become a differentiated and competitive solution in the sustainable coatings sector.

These observations reveal the need for integrative studies that link green synthesis, coating formulation, and performance evaluation under realistic conditions. Advancing sustainable multifunctional coatings requires a holistic understanding of how synthesis-derived nanoparticle properties translate into self-cleaning, antifungal, and photocatalytic behaviors on construction-relevant surfaces. To address these gaps, the present study synthesizes TiO_2_ nanoparticles using *Cymbopogon citratus* lemongrass extract, characterizes their structural and morphological properties, incorporates them into water-based acrylic coatings at controlled loadings, and evaluates their self-cleaning, photocatalytic, and antifungal performance on fiber-cement substrates.

## 2. Materials and Methods

### 2.1. Materials

Fresh lemongrass leaves (*Cymbopogon citratus*) were collected in the Gambote district, Arjona, Bolivar (Colombia). Titanium isopropoxide (C_12_H_28_O_4_Ti, 95%) and methylene blue dye (C_16_H_18_ClN_3_S) were purchased from Sigma-Aldrich (Burlington, MA, USA). Absolute ethyl alcohol (C_2_H_5_OH, 99.5%) was supplied by Merck KGaA (Darmstadt, Germany). Acrylic paint and primer were obtained from Corona © (Bogotá, Colombia). Distilled water was used as the solvent in all tests. *Aspergillus niger* and *Penicillium* spp. Strains were used for the antifungal activity tests.

### 2.2. Preparation of the Cymbopogon citratus Extract

Fresh *Cymbopogon citratus* leaves were sorted, cut, and washed with distilled water to remove impurities. The biomass was dried in an oven at 60 °C for 6 h to remove moisture and then ground. The extract was obtained by the infusion method, in which 100 g of dried biomass was added to 600 mL of distilled water and heated to 80 °C for 20 min. After this process, it was filtered to separate the solid residues [7].

### 2.3. Synthesis of TiO_2_ Nanoparticles

**Figure 1:**
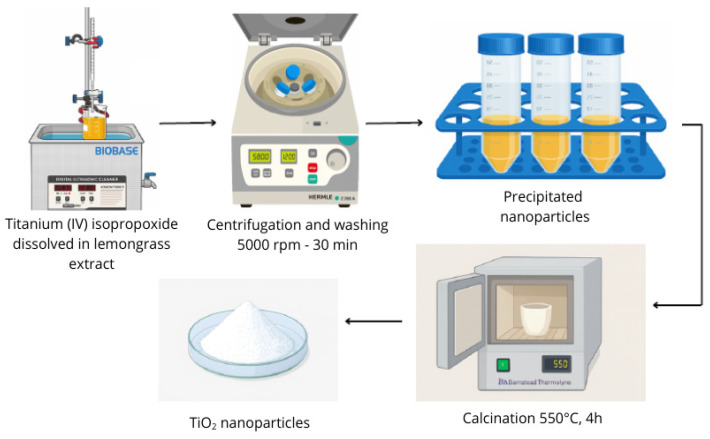
Preparation of TiO_2_ nanoparticles by green chemistry synthesis.

For the synthesis, 100 mL of *Cymbopogon citratus* extract was mixed with 20 mL of titanium (IV) isopropoxide via dropwise addition under constant stirring. Subsequently, the mixture was subjected to ultrasonication at 60 Hz and 45 °C for 30 min. The resulting solution was centrifuged at 5000 rpm for 30 min to separate the formed nanoparticles. The precipitated nanomaterials were washed twice with distilled water and once with ethanol to remove any remaining unreacted precursor. Finally, the obtained nanoparticles were placed in a crucible and calcined at 550 °C for 4 h [7]. Figure 1 displays a schematic of the synthesis process.

### 2.4. Characterizations

The morphology and elemental composition of the TiO_2_ nanoparticles were analyzed using a scanning electron microscope (SEM) coupled with energy-dispersive X-ray spectroscopy (EDS), employing a Thermo Fisher Scientific (ThermoFisher Scientific, Waltham, MA, USA) Scios 2 LoVac Dual Beam field emission microscope, equipped with an UltraDry 129 eV, 30 mm^2^ EDS microanalysis system (SDBX-30PM-B). The analysis was performed with resolutions of 0.7 nm at 30 kV in STEM mode and 1.4 nm at 1 kV in FESEM mode [21]. The transmission electron microscopy (TEM) analysis was performed using a Tecnai F20 Super Twin TMP (FEI, Hillsboro, OR, USA, EE. UU), operated at an acceleration voltage of 200 kV with an emission gun and a beam current of 108.6 µA, equipped with diffraction mode. The crystalline structure was analyzed and evaluated by X-ray diffraction (XRD) using a Malvern-PANalytical Empyrean 2012 diffractometer (Malvern Panalytical, Malvern, UK), with Cu-Kα radiation (λ = 1.541 Å) at 45 kV and 40 mA, in a 2*θ* range of 10° to 80°, with a step of 0.05° and a counting time of 50 s per step, identifying the crystalline phases present in the samples [22]. Diffuse reflectance spectroscopy (UV–vis DRS)/powder was used to determine the band gap of the synthesized TiO_2_ nanoparticles, utilizing a UV–Vis diffuse reflectance Spectrophotometer, Evolution-600 (Thermo Scientific, Waltham, MA, USA), with a wavelength ranging from 200 to 800 nm, scanning speed of 240 nm/min, and BaSO4 as reference.

### 2.5. Modification of the Acrylic Coating

**Figure 2:**
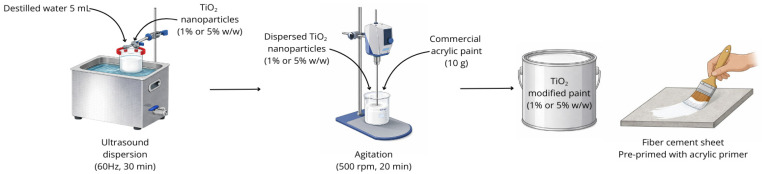
Schematic illustration of the preparation of acrylic coatings modified with TiO_2_ nanoparticles.

A commercial water-based coating (PARAGUAS^®^ MULTIPROPÓSITO, Corona ©) was used to incorporate TiO_2_ nanoparticles at 1% and 5% *w*/*w*. Nanoparticles were first dispersed in 5 mL of distilled water via ultrasound (60 Hz, 30 min), then mixed with 10 g of the acrylic paint under agitation (500 rpm, 20 min) [23]. The mixture was applied to 10 × 10 cm fiber cement sheets pre-primed with acrylic primer (ACRILCOR 50, Corona ©). After drying, three coats were applied, followed by testing for self-cleaning via methylene blue photodegradation and contact angle assays. Figure 2 illustrates the experimental methodology used to modify the acrylic coating with TiO_2_ nanoparticles.

### 2.6. Methylene Blue Photodegradation Test

The photocatalytic degradation activity of the modified coating was evaluated using methylene blue dye (MB) at concentrations of 5, 10, and 20 mg/L as a reference contaminant [8]. A 200 µL drop was applied to each coated sheet and subsequently exposed to sunlight under a Solar Spectrum Pro system (LED, visible light, UVA and UVB, 55,000 lux) for 24 h.

Degradation was analyzed by color variation using ImageJ version 1.54g (Color Histogram plugin), converting the RGB values to the CIELAB color system. In this system, L* represents the luminosity from white to black, a* the scale from red to green, and b* the values of blue and yellow. The *AM* degradation analysis focused on the variation in the b* coordinate, the reference parameter for the blue color, comparing the initial values (b*(0 h)) with those obtained after 4 h (b*(4 h)) and 24 h (b*(24 h)) of exposure. The results were compared with a control sheet containing an unmodified coating [24]. The color removal efficiency of RhB was calculated using Equations (1) and (2), considering that a material can be photocatalytic if it meets the efficiency criteria *AM* (4 h) > 20% and *AM* (24 h) > 50% [25].

(1) $${AM}_{4h}\%=\left[\frac{\left(b^{*}\left(0\;h\right)-b^{*}\left(4\;h\right)\right)}{b^{*}\left(0\;h\right)}\right]\times 100,$$

(2) $${AM}_{24h}\%=\left[\frac{\left(b^{*}\left(0\;h\right)-b^{*}\left(24\;h\right)\right)}{b^{*}\left(0\;h\right)}\right]\times 100,$$

### 2.7. Methylene Blue Photodegradation Mechanism

The photodegradation of methylene blue (MB) using TiO_2_ nanoparticles under simulated irradiation is explained by a sequence of photocatalytic reactions induced by the semiconductor properties [26]. First, the photon absorption with energy equal to or greater than the band gap of the TiO_2_ anatase phase (*E_g_* ≈ 3.2 eV) promotes electrons from the valence band to the conduction band, generating electron–hole pairs (Equation (3)).

(3) $${TiO}_{2}+hv\to e_{\left(CB\right)}^{-}+h_{\left(VB\right)}^{+}$$

These charge carriers migrate to the photocatalyst’s surface, where they participate in redox reactions. The holes $h_{\left(VB\right)}^{+}$ oxidize adsorbed water or hydroxyl ions, producing highly reactive hydroxyl radicals (Equations (4) and (5)):

(4) $$h_{\left(VB\right)}^{+}+H_{2}O\to \cdot OH+H^{+}$$

(5) $$h_{\left(VB\right)}^{+}+OH^{-}\to \cdot OH$$

Simultaneously, the excited electrons in the conduction band $e_{\left(CB\right)}^{-}$ reduce the dissolved oxygen on the coating surface, generating superoxide radicals (Equation (6)), which can subsequently transform into other oxidizing species, such as hydrogen peroxide and additional hydroxyl radicals (Equations (6)–(9)):

(6) $$e_{\left(CB\right)}^{-}+O_{2}\to O_{2}^{\cdot -}$$

(7) $$O_{2}^{\cdot -}+H^{+}\to {HO}_{2}^{\cdot }$$

(8) $$2{HO}_{2}^{\cdot }\to {H_{2}O}_{2}+O_{2}$$

(9) $${H_{2}O}_{2}+e_{\left(CB\right)}^{-}\to \cdot OH+OH^{-}$$

Reactive oxygen species ($\cdot OH,\;O_{2}^{\cdot -}$), along with the gaps in the VB, attack the methylene blue molecules adsorbed on the coating surface, promoting their oxidation. This leads to the formation of degraded intermediates that subsequently fragment into simpler compounds, such as carbon dioxide and water (Equations (10) and (11)):

(10) $$\cdot OH+AM\to {CO}_{2}+H_{2}O+\;oxidized\;intermediates\;$$

(11) $$O_{2}^{\cdot -}+AM\to {CO}_{2}+H_{2}O+oxidized\;intermediates$$

### 2.8. Contact Angle Determination

The hydrophobic capacity was evaluated using the sessile droplet method, measuring the static contact angle [27]. A 10 µL drop of distilled water was placed on the surface, images were captured with an electron microscope at a magnification of 1600×, and the contact angle was determined using the ImageJ version 1.54g (DropSnake Plugin) [28].

### 2.9. Antifungal Property Evaluation

Antifungal activity was assessed against *Aspergillus niger* and *Penicillium*, which are characterized by their ability to grow on surfaces such as walls. Potato dextrose agar (PDA) was used as the culture medium, and the Poisoned Food technique described by Das et al. [29] was applied. Nanoparticles were dispersed in 10 mL of deionized water to obtain final agar concentrations of 1 and 3 mg/mL, and then ultrasonicated for 30 min. It was incorporated into 50 mL of PDA and sterilized in an autoclave at 121 °C for 1 h. After this time, 25 mL of the modified culture medium was poured into Petri dishes (90 mm × 15 mm) and allowed to cool for subsequent inoculation [30]. Fungal inoculation was performed using the stab inoculation technique, employing a straight loop to transfer a portion of the mycelium into the modified culture medium. Incubation was carried out at room temperature and observations were made at 48 and 120 h. The results were compared with negative controls (PDA medium without nanoparticles) and positive controls (fluconazole). To avoid interference from the coating matrix in the results, antifungal activity was evaluated directly on the nanoparticles.

## 3. Results

The following section presents and analyzes the experimental results obtained in this study. First, the scanning electron microscopy coupled with energy-dispersive X-ray spectroscopy (SEM-EDS), transmission electron microscopy (TEM), UV–vis DRS, and X-ray diffraction (XRD) analyses is reported, which allowed us to determine the morphology, elemental composition, size, band gap, and crystalline structure of the TiO_2_ nanoparticles synthesized via green chemistry. Subsequently, the results for the functional properties of the acrylic coating modified with different nanoparticle concentrations are presented, including its photocatalytic activity (self-cleaning), hydrophobicity (measured by contact angles), and antifungal performance against *Aspergillus niger* and *Penicillium* spp. These properties are discussed in the context of their potential application to construction surfaces, particularly concrete roofs and lightweight asbestos–cement coverings, where improvements in dirt resistance, waterproofing capacity, and microbial colonization prevention represent a significant advance in sustainability and coating durability.

### 3.1. SEM, TEM, and EDS Analysis

**Figure 3:**
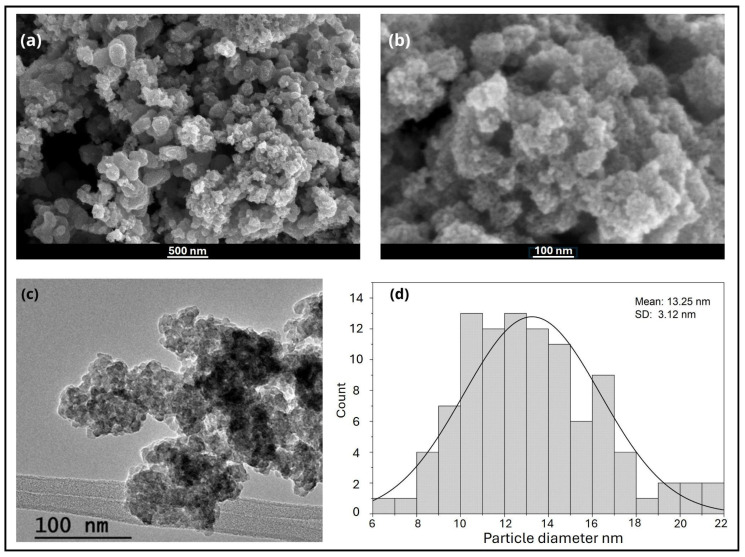
SEM images of TiO_2_ nanoparticles synthesized using lemongrass extract at (**a**) 50,000× magnification, (**b**) 250,000× magnification, (**c**) TEM image of TiO_2_ nanoparticles, and (**d**) particle size distribution.

**Figure 4:**
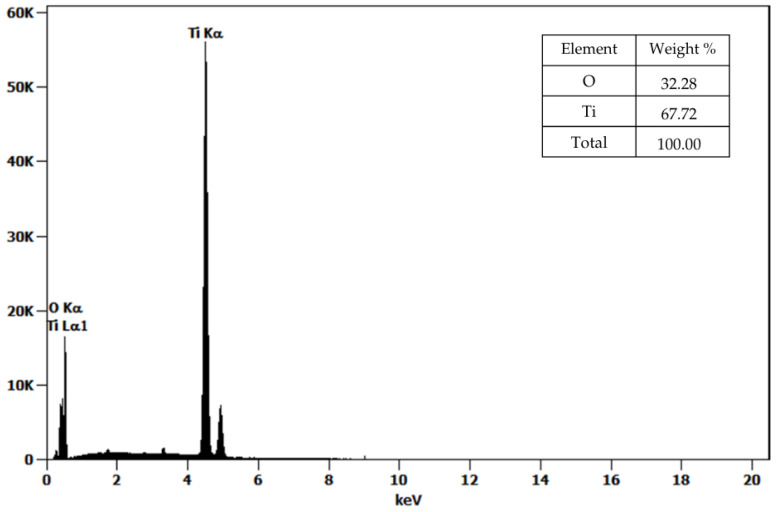
Energy-dispersive X-ray spectroscopy (EDS) spectra and elemental composition TiO_2_ nanoparticles.

The SEM micrographs revealed that the TiO_2_ nanoparticles synthesized via green chemistry using *Cymbopogon citratus* extract exhibited an irregular quasi-spherical morphology with a marked tendency to form compact agglomerates, which is characteristic of nanoscale materials due to high surface energy and van der Waals interactions (Figure 3a,b). The average particle size was estimated from TEM images (Figure 3c) using ImageJ version 1.54g software. From this measurement, an average nanoparticle size of 13.25 ± 3.12 nm was obtained, confirming the successful formation of TiO_2_ nanoparticles within the expected nanometric range. EDS analysis (Figure 4) confirmed the presence of titanium (Ti) and oxygen (O) as the dominant elements, with atomic percentages of 41.21% Ti and 58.79% O, consistent with the stoichiometric composition of TiO_2_ and indicating the absence of significant impurities from the synthesis process. These findings are in close agreement with those reported by Sahraoui et al. [31], where TiO_2_ nanoparticles synthesized using *Eucalyptus globulus* extract also presented quasi-spherical agglomerates and particle sizes in the range of 13–15 nm, comparable to commercial TiO_2_ (10–13 nm). Similarly, both studies show Ti and O as the main elemental peaks, although the work by [31] reported a minor carbon signal attributed to organic residues from the plant extract. In contrast, the absence of detectable carbon in our EDS spectra suggests more efficient decomposition of organic components during thermal treatment at 550 °C, likely resulting in higher-purity TiO_2_ nanoparticles. This interpretation is supported by thermogravimetric analyses reported for *Cymbopogon citratus*-derived matrices, which show that the major mass loss associated with the degradation of organic constituents—such as phenolics, terpenoids, cellulose, and hemicellulose—occurs mainly below ~500 °C, with negligible to residual mass at higher temperatures [7,32]. Consistently, TG/DTG profiles of lemongrass essential oil show that the volatile fraction is lost at much lower temperatures (typically completing evaporation below ~200 °C), indicating that these organics cannot persist under high-temperature calcination [33]. Therefore, calcination at 550 °C is sufficient to ensure the complete removal of organic residues originating from the plant extract. Similar behavior has been widely reported in green synthesis routes for TiO_2_ nanoparticles using plant extracts, where calcination temperatures above 500 °C effectively eliminate carbonaceous species and yield phase-pure oxide materials [34].

On the other hand, the study by Parvez et al. [35] also described spherical nanoparticles whose average size varied with the degree of co-doping. They noted that particle aggregation is strongly influenced by surface charge and pH, factors that control electrostatic repulsion between particles. The agglomeration observed in our samples may similarly arise from insufficient electrostatic stabilization during the green synthesis, as no surfactants or charge-modifying agents were used, consistent with the eco-friendly approach adopted.

Furthermore, these results align with the observations determined from Korkmaz et al. [36], who reported spherical, clustered TiO_2_ nanoparticles synthesized from *Azadirachta indica* and *Calotropis gigantea* extracts with diameters ranging from 21 to 29 nm, slightly larger than those reported in the present work. The smaller mean particle size obtained here (13.25 nm) could be attributed to the specific composition of the lemongrass extract, which is rich in flavonoids and aldehydes, and may act as effective capping and reducing agents, limiting excessive particle growth. Additionally, ref. [36] reported minor impurities, such as Cl and K residues, whereas our EDS data indicated a cleaner surface composition, reinforcing the efficiency of the washing and calcination steps in removing unreacted precursors.

Unlike the composite TiO_2_-based nanostructures discussed by Moeini-Eghbali et al. [37], where Ti was part of a Fe_3_O_4_/TiO_2_–Ni nanocomposite with multiple elements confirmed by EDX mapping, the nanoparticles synthesized in this study exhibit a pure TiO_2_ composition, as expected for single-phase oxide nanoparticles. The uniform distribution of Ti and O displayed in the EDS mapping (Figure 4) corroborates the homogeneity of the synthesized nanomaterials and supports the successful formation of nanocrystalline TiO_2_ without incorporation of secondary metallic species.

The morphology and composition obtained in this study are consistent with previously reported green synthesis routes, confirming the effectiveness of lemongrass extract as a capping and stabilizing agent. The smaller average size and absence of detectable impurities highlight the purity and structural integrity of the nanoparticles produced under mild synthesis conditions, demonstrating that sustainable methods can yield TiO_2_ nanostructures comparable to, or even superior in quality to, those produced by conventional or other plant-assisted approaches.

### 3.2. UV–Vis DRS

**Figure 5:**
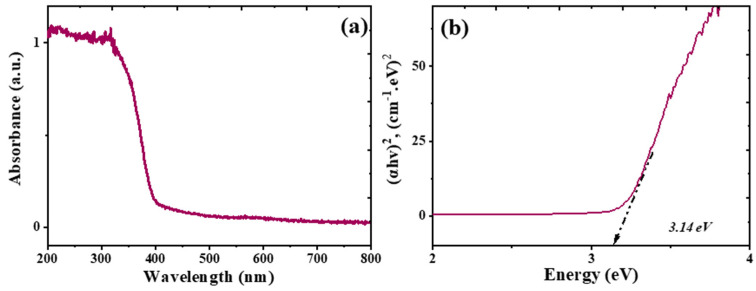
Optical properties of TiO_2_ nanocomposites synthesized by green chemistry using lemongrass extract: (**a**) UV–vis DRS spectra; (**b**) Tauc plot study for the band gap (eV) estimation.

Figure 5a shows the UV–Vis absorption spectra of TiO_2_ nanoparticles synthesized in the presence of the lemon grass extract. Furthermore, the Tauc relation (Equation (12)) was employed to determine the band gap energy of these nanoparticles.

(12) $${\left(ahv\right)}^{1/n}=B\left(hv-E_{g}\right);\;\;hv=\frac{1240}{\lambda };\;\;\alpha =2.3\frac{A}{t}$$

where *n* = 0.5, *α* is the absorption coefficient for each wavelength, *hv* is the incident photon energy (eV), *B* is an energy-dependent constant that is known as the band-tailing parameter, *λ* is the wavelength (nm), *E_g_* is the optical energy gap (eV), *A* is the absorbance and *t* is the thickness (1 cm). Figure 5b illustrates this result, obtaining a band gap of 3.14 eV for the synthesized TiO_2_ nanoparticles, which compares very well with the value reported in the literature for commercial P25 TiO_2_ nanomaterials at about 3.2 eV [38,39].

### 3.3. XRD Analysis

**Figure 6:**
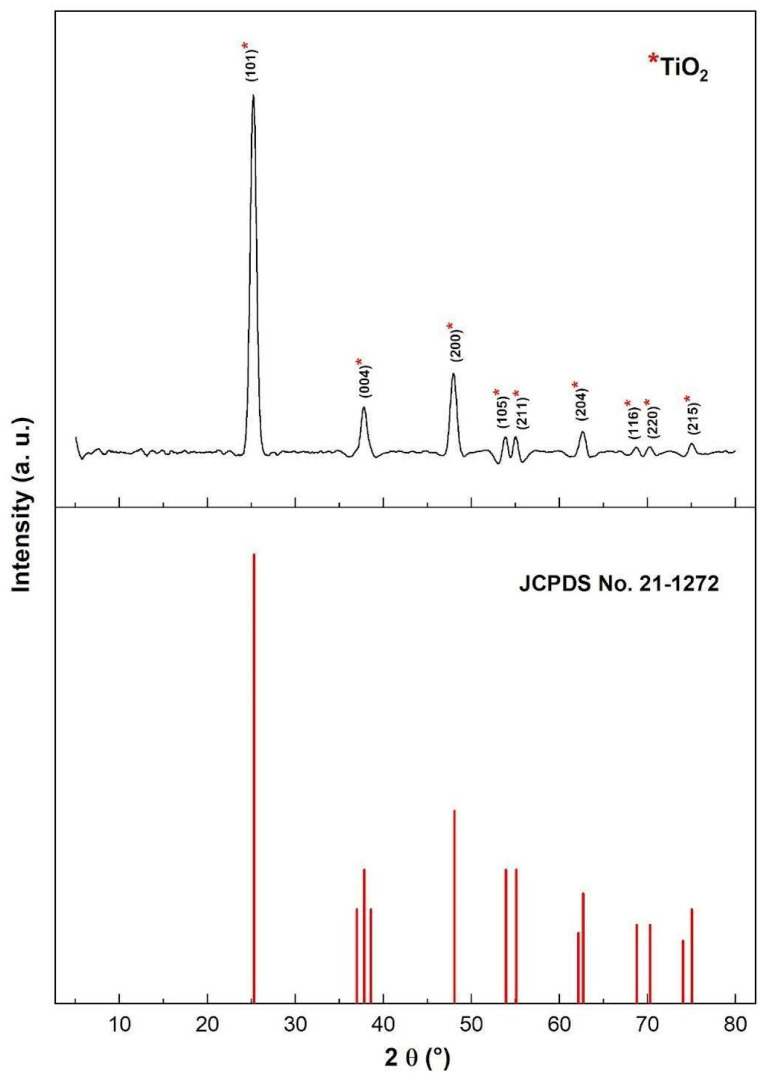
XRD pattern of TiO_2_ anatase green synthesized nanoparticles.

The X-ray diffraction (XRD) pattern of the green-synthesized TiO_2_ nanoparticles is presented in Figure 6. The diffraction peaks located at 2*θ* = 25.19°, 37.75°, 47.94°, 53.82°, 54.97°, 62.75°, 68.63°, 70.15°, and 75.09° correspond to the crystallographic planes (101), (004), (200), (105), (211), (204), (116), (220), and (215), respectively, matching the reference card JCPDS 21-1272 [22] for the anatase phase of TiO_2_. The absence of secondary diffraction peaks confirms the formation of a highly pure crystalline structure, with no detectable traces of rutile or brookite phases. The intense reflection at 2*θ* = 25.19°, corresponding to the (101) plane, indicates that anatase is the predominant phase, which is well known for its superior photocatalytic performance. The broadening of the diffraction peaks, particularly those observed around 25° and 47°, reveals the nanocrystalline nature of the synthesized material. The average crystallite size, calculated using the Debye–Scherrer equation (Equation (13)), was 11.13 nm, confirming the successful synthesis of TiO_2_ nanoparticles with dimensions in the nanometric range.

(13) $$D=\frac{k\lambda }{\beta cos\theta }$$

where *D* is the average crystallite size, *θ* represents the Bragg angle, *λ* denotes the wavelength of the X-ray radiation (1.541 Å), *β* represents the full width at half maximum (FWHM) of the diffraction peak, and k is a constant (0.9). These findings are consistent with previous reports by Liu et al. [40], who also observed that green-synthesized TiO_2_ nanoparticles exhibit pronounced anatase reflections and high phase purity.

These results are in agreement with those reported by [41] where TiO_2_ nanoparticles biosynthesized using plant extracts also exhibited pure anatase phase with peaks at approximately 25.26°, 37.84°, 48.02°, 54.99°, 62.55°, 69.77°, 75.20°, and 82.77°. Both studies confirm that biologically assisted synthesis can produce phase-pure anatase TiO_2_ without secondary impurities. However, the crystallite size in the present study (11.13 nm) is considerably smaller than the ≈39 nm reported by [35]. This difference can likely be attributed to the distinct organic composition of the lemongrass extract, which is rich in phenolic and aldehydic compounds that act as effective capping and stabilizing agents, limiting particle growth during hydrolysis and calcination.

The XRD results also correspond closely with those obtained by [36] where pure TiO_2_ nanoparticles prepared via a green route showed sharp and well-defined anatase peaks at 25.31°, 37.92°, 48.00°, 54.54°, 55.04°, 62.72°, and 75.29°—values almost identical to those of the present work. In both cases, the strongest diffraction line corresponds to the (101) plane, confirming that the anatase phase is dominant. Nevertheless, the average crystallite size reported for pure TiO_2_ in [36] (6.8 nm) was slightly smaller than in our synthesis (11.13 nm). Such variation may stem from differences in precursor chemistry and thermal treatment: in our case, calcination at 550 °C for 4 h was optimized to ensure phase stability and purity rather than minimal size.

Compared with the *Eucalyptus globulus*-based synthesis described by [35], our results show similar diffraction behavior—ten well-defined peaks indexed to the anatase structure and the absence of impurity signals. In both studies, the (101) plane exhibited the highest intensity, highlighting the formation of highly crystalline anatase TiO_2_. Yet, ref. [4] reported minor rutile contributions in their samples (≈20%), whereas no rutile reflections were detected in the present case, suggesting that the synthesis route using lemongrass extract and moderate calcination temperature effectively inhibits the anatase-to-rutile transformation. This stabilization can be attributed to the organic components of the extract acting as surface ligands that delay phase transition during heating.

In contrast, the XRD data for the Fe_3_O_4_/TiO_2_–Ni nanocomposite analyzed by [37] exhibited multiple reflections corresponding to both Fe_3_O_4_ and TiO_2_ phases, confirming the coexistence of magnetic and semiconductor structures. While their TiO_2_ peaks at 25.2°, 36.9°, 48°, 55°, 62°, 68.9°, 70.3°, and 74.9° are consistent with the anatase lattice, the composite nature of their material and the additional Ni and Fe peaks distinguish it clearly from our single-phase TiO_2_ system. The absence of any extra peaks in our diffractogram underscores the high purity and homogeneity of the nanoparticles obtained by green synthesis without metallic dopants.

The XRD analysis confirms that the TiO_2_ nanoparticles synthesized using *Cymbopogon citratus* extract possess phase-pure anatase structure, excellent crystallinity, and nanometric size (~11 nm). The combination of small crystallite size and stable anatase phase is advantageous for photocatalytic applications, as it enhances surface area and charge separation efficiency. Compared to other plant-mediated syntheses reported in the literature, the present route yields smaller crystal sizes and higher phase purity, validating the effectiveness of lemongrass-assisted green synthesis as a sustainable and reliable alternative for producing functional TiO_2_ nanomaterials.

### 3.4. Photocatalytic Degradation of Methylene Blue Under Simulated Sunlight

**Figure 7:**
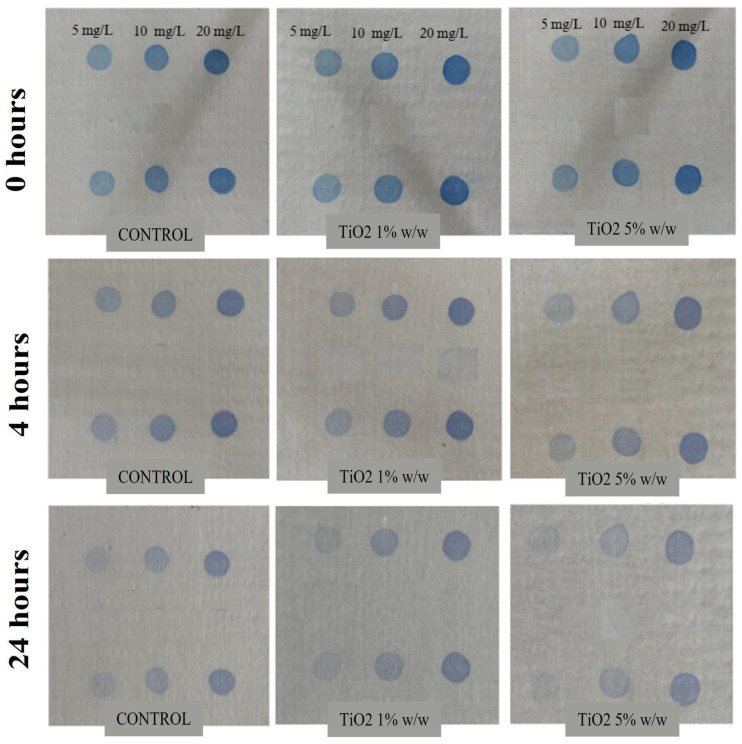
Photodegradation test for modified coating with TiO_2_ nanoparticles.

Figure 7 illustrates the color evolution of methylene blue (MB) applied on fibrocement plates coated with conventional acrylic paint (control) and with TiO_2_-modified acrylic coatings containing 1 wt% and 5 wt% of nanoparticles. The samples were exposed to simulated sunlight for 0, 4, and 24 h at MB concentrations of 5, 10, and 20 mg L^−1^. In the control, a gradual fading of the blue color was observed over time; however, visible stains persisted even after 24 h, indicating only partial degradation. This limited photocatalytic response may be attributed to the minor TiO_2_ content already present in the commercial formulation. In contrast, the coatings modified with TiO_2_ nanoparticles exhibited a markedly higher discoloration rate. In particular, the 5 wt% TiO_2_ coating almost completely eliminated MB stains after 24 h at the lowest dye concentration (5 mg L^−1^), demonstrating a superior photocatalytic self-cleaning effect.

This visual behavior is corroborated by the quantitative results summarized in Table 1, which show a substantial increase in photocatalytic efficiency with nanoparticle incorporation. For the control coating, the degradation efficiency ranged from 30 to 47% at 4 h and 49–77% at 24 h, reflecting limited photoactivity. When 1 wt% TiO_2_ was added, the degradation efficiency increased to 71.9% (4 h) and 89.1% (24 h) at 5 mg L^−1^ MB. At higher dye concentrations (20 mg L^−1^), the efficiency decreased to 38.2% and 57.1%, respectively, likely due to surface saturation and competition for active sites on TiO_2_. The best performance was observed with the 5 wt% TiO_2_ coating, which reached 93.3% degradation after 4 h and 96.4% after 24 h at 5 mg L^−1^. Nevertheless, at 20 mg L^−1^ MB, efficiency declined to 49.9% (4 h) and 67.7% (24 h), consistent with reduced reactive oxygen species generation under high pollutant loading [42]. According to Guo et al., the nanoparticle studied exhibits sustained degradation activity over time as the dye concentration decreases, exceeding the 50% efficiency threshold [25].

The temporal evolution of the photocatalytic response further indicates a saturation-like behavior at low dye concentrations and higher TiO_2_ loadings. As reported by Fujishima et al. [43], when the concentration of organic pollutants decreases, the photocatalytic rate becomes limited by reactant availability rather than by the number of active sites, leading to marginal efficiency gains at extended irradiation times. In the case of TiO_2_-based coatings for construction materials, Chen and Poon [44] reported that increasing TiO_2_ content beyond an optimal threshold does not necessarily improve photocatalytic performance and may adversely affect optical and functional properties. Similarly, ref [45] demonstrated that excessive TiO_2_ loading in polymeric matrices promotes nanoparticle agglomeration and light-scattering effects, reducing effective photon penetration and enhancing electron–hole recombination. Therefore, the subtle visual differences observed between 4 h and 24 h of irradiation for the 5 wt% TiO_2_ coating in Figure 7 are indicative of a near-complete degradation regime rather than insufficient photocatalytic activity, supporting the suitability of this TiO_2_ loading for self-cleaning acrylic coatings.

These findings align with the trends reported in the literature, though with notable contextual differences. For example, Sangeetha et al. (2024) [41] achieved degradation rates of 97–99% for boron–cerium co-doped TiO_2_ nanoparticles under visible light within 150 min. Similarly, Aghababaei et al. [46] reported efficiencies up to 87.45% for Ce-doped TiO_2_–Fe_3_O_4_–Cu nanocomposites under optimized UV conditions. These materials benefited from band gap narrowing and electron–hole recombination suppression induced by doping or hybridization with magnetic phases. In another study, Vijayarohini and co-workers evaluated the application of TiO_2_ nanoparticles doped with nickel atoms as an additive for commercial acrylic water paint [38]. In this work, the photocatalytic self-cleaning efficiency and surface disinfection of TiO_2_-Ni nanomaterials were analyzed using p-xylene (1 ppm) as an air pollutant under simulated indoor illumination. They observed an 87% of p-xylene decomposition after using TiO_2_-Ni nanoparticles (N/Ti: 0.5–2). In contrast, the present study demonstrates that high photocatalytic performance (up to 96.4%) can also be achieved for MB removal using undoped TiO_2_ synthesized via a green route, incorporated into a commercial water-based acrylic matrix—a far less complex and more environmentally friendly system.

Compared to [47], where TiO_2_-reinforced nylon nanofibers reached 88.68% MB degradation after 60 min under UV light, the TiO_2_–acrylic coatings in this work exhibited comparable or superior activity under simulated sunlight and over extended exposure periods. The higher efficiency obtained here can be attributed to the uniform dispersion of anatase-phase TiO_2_ nanoparticles (11 nm average crystallite size) within the acrylic matrix, which facilitates optimal light interaction and improved generation of hydroxyl radicals. Moreover, the coating’s hydrophobic yet photocatalytically active surface enhances self-cleaning by limiting dye adsorption and promoting contaminant detachment upon oxidation.

In another contribution, Islam and collaborators studied the development of a photocatalytic paint containing TiO_2_ nanoparticles (1–10 wt%) for the photodegradation of organic dye pollutants (6 ppm), such as methylene blue (MB) and methyl orange (MO) [48]. After 120 min of light irradiation, it was determined that 90% of MO removal and 80% of MB photodegradation occurred with the highest concentration of TiO_2_ nanoparticles (10 wt%), suggesting the potential of this photocatalytic nanomaterial for self-cleaning applications. Additionally, Wu and co-workers evaluated the incorporation of TiO_2_ nanoparticles (5% wt) for self-cleaning traffic marking coatings [49]. In this work, they studied the photocatalytic degradation of the organic dyes methylene blue (MB), methyl orange (MO), and Rhodamine-B (RhB) under UV irradiation. They observed a time-dependent decolorization effect for all organic dyes, with the effect more pronounced for the MO dye, resulting in near-complete degradation of this molecule after 12 h of UV treatment, compared with the MB and RhB dyes under the same experimental conditions. This can be attributed to electrostatic interactions between the immobilized TiO_2_ nanoparticles and the organic pollutants, as MO is a simple anionic dye, MB is a simple cationic dye, and RhB is a complex cationic molecule. Furthermore, RhB has hydrophobic aromatic rings in its chemical structure, which can affect the kinetics of the photodegradation process, and can act as a sensitizer of the TiO_2_ nanoparticle, providing electrons into the conduction band of the photocatalytic nanomaterial.

Unlike most photocatalytic studies conducted on inert substrates such as glass or polymer films, this work evaluated the coatings directly on asbestos–cement roofing plates, a material widely used in urban environments of developing countries like Colombia. This substrate choice is particularly relevant because TiO_2_-based coatings can extend the service life of asbestos–cement surfaces by reducing dirt accumulation, improving water repellency, and preventing the colonization of microorganisms—issues which are commonly reported in tropical climates [50,51]. Therefore, beyond the high degradation efficiency achieved, the present study demonstrates the practical feasibility of incorporating TiO_2_ nanoparticles into commercial coatings for self-cleaning, hydrophobic, and antifungal surface protection in real construction applications.

### 3.5. Contact Angle Test

**Figure 8:**
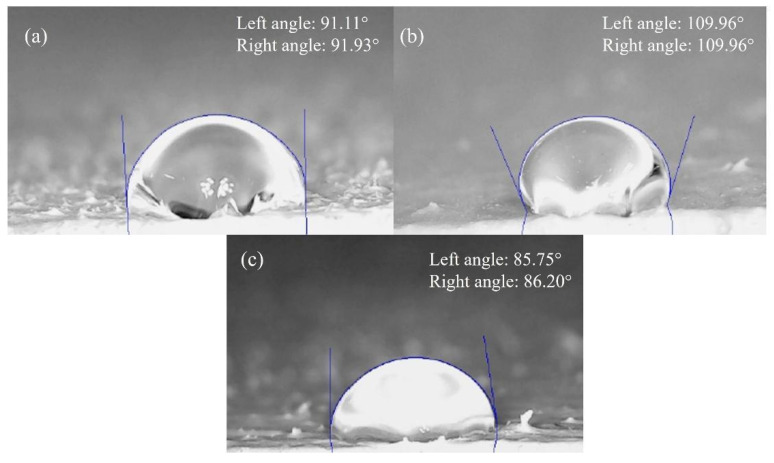
Droplet profiles for coating modified with TiO2 nanoparticles (**a**) 1%, (**b**) 5% and (**c**) control.

The contact angle results for water droplets are summarized in Table 2. Measurements were obtained using ImageJ version 1.54g software with the DropSnake plugin, which applies flexible B-spline curves to fit the actual droplet profile and accurately determine contact points [28]. Figure 8 displays representative images of the sessile-drop measurements. Average contact angles of 80.4°, 92.03°, and 104.25° were recorded for coatings containing 0%, 1%, and 5 *w*/*w*% TiO_2_, respectively.

These results clearly demonstrate a progressive increase in contact angle with increasing TiO_2_ nanoparticle concentration. The incorporation of TiO_2_ into the acrylic matrix alters its surface free energy, reducing wettability and thereby increasing hydrophobicity. This modification improves the coating’s self-cleaning capacity, as higher contact angles facilitate the rolling-off of water droplets that remove surface contaminants. These observations are consistent with previous reports on coatings of different chemical nature modified by the addition of TiO_2_ nanoparticles [52,53,54]. The calculated surface free energy (−∆*G_sl_*), determined using the Young–Dupré equation (Equation (14)), confirmed this trend [28].

(14) $$-∆G_{sl}=\gamma l\left(1+cos\theta \right)$$

where *θ* is the contact angle and *γl* is the surface tension of water, which in this case will have a value of 72.8 mN/m [55]. Table 3 shows the calculated values corresponding to the surface energy. Using the surface energy values, the percentage improvement with respect to the concentration of nanoparticles was calculated in comparison to the coating without nanoparticles using Equation (15).

(15) $$\%\;improvement=\frac{-∆G_{sl}\;\%0-(-∆G_{sl}m)}{-∆G_{sl}\;0\%}\times 100$$

Compared to the unmodified coating, the improvement in surface energy was 11.6% for 1 wt% TiO_2_ and 30.9% for 5 wt% TiO_2_ (Table 3). These findings highlight that TiO_2_ nanoparticles significantly enhance the hydrophobic character of the commercial acrylic coating, resulting in superior water repellency and self-cleaning behavior.

Here, $-∆G_{sl}m$ refers to the surface energy of each % *w*/*w* of the coatings. The percentage improvement for titanium dioxide at a concentration of 1% *w*/*w* was 11.6%, and for titanium dioxide at a concentration of 5% *w*/*w* it was 30.9%.

TiO_2_ nanoparticles significantly enhance self-cleaning properties of the commercial acrylic coating by modifying its surface energy, thereby reducing the adhesion of pollutant particles. The resulting increase in surface hydrophobicity promotes the movement of water droplets, which efficiently carry away adhered dirt and reduce the need for cleaning procedures [56].

The increase in contact angle observed here is consistent with previous studies on TiO_2_-based composite coatings. For instance, Sajadinia et al. [55] reported superhydrophobic coatings on concrete substrates containing mixed nanoparticles (SA/TiO_2_/CuO/SiO_2_) with water contact angles (WCAs) of up to 159° and sliding angles as low as 3°, due to the combined effects of low surface energy and micro/nano surface roughness. Similarly, Liu et al. [57] achieved WCAs of 154–160° in Mg-modified TiO_2_ nanostructures, highlighting that hydrophobic surface modification and improved nanoparticle dispersion are key to reducing surface energy and preventing water infiltration.

Although the hydrophobicity achieved in our system (104.25°) does not reach the superhydrophobic range (>150°), it is remarkable considering that the present study employed undoped TiO_2_ nanoparticles synthesized by a green route, incorporated into a commercial water-based acrylic paint—a simpler and more environmentally benign system compared to chemically functionalized or multi-component coatings. Moreover, the obtained WCA values exceed those reported for pure TiO_2_-containing nanofiber composites, in which TiO_2_ typically enhances hydrophilicity rather than hydrophobicity. For example, ref. [47] observed a decrease in contact angle (from 63° to ~40°) upon adding TiO_2_ to nylon nanofibers, attributed to the polar surface character of TiO_2_. In contrast, in our acrylic matrix, TiO_2_ nanoparticles likely induce surface restructuring and micro-roughness that, together with the hydrophobic polymeric binder, yield the opposite effect—an increase in contact angle and reduced wettability.

Comparable trends were also reported by Yang et al. [58], who found that Fe–TiO_2_–graphene composite coatings exhibited a WCA of 142.8° with pronounced self-cleaning and water repellent properties. The authors noted that coatings with larger contact angles displayed better dirt removal and slower aging, confirming that hydrophobicity contributes directly to long-term surface durability. Similarly, the increase in hydrophobicity observed in our TiO_2_–acrylic coatings suggest improved resistance to fouling and moisture penetration, which is critical for maintaining the mechanical and optical integrity of exterior coatings exposed to humid tropical environments.

In the broader context of application development, the enhanced hydrophobicity achieved here has significant implications. When applied to asbestos–cement and concrete roofing surfaces, such coatings can extend the service life of structures by preventing water absorption, minimizing microorganism colonization, and facilitating dirt removal—issues which are particularly relevant in urban areas of developing countries such as Colombia, where asbestos–cement roofs remain widespread [50,51].

### 3.6. Evaluation of Antifungal Properties

**Figure 9:**
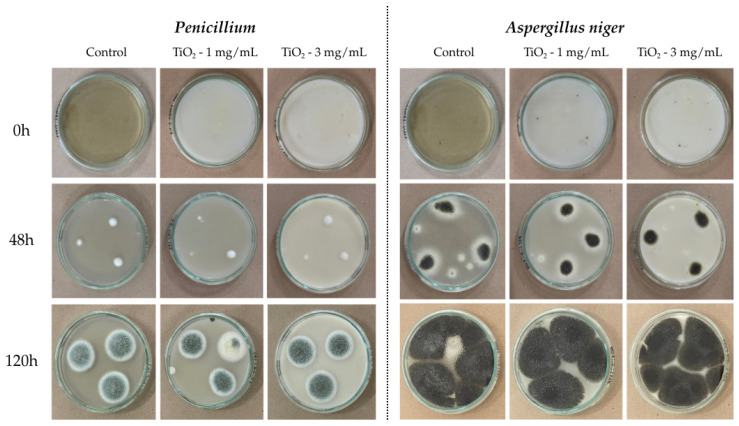
Test of antifungal activity against *Penicillium* and *Aspergillus niger*.

The results in Figure 9 indicate that the fungus *Aspergillus niger* exhibited abundant growth, with a deep black mycelium characteristic of this species. In contrast, *Penicillium* showed more controlled growth and green mycelia.

TiO_2_ nanoparticles reduced the average growth area of the *Penicillium* fungus compared to the control. Assays with the *Aspergillus niger* fungus showed almost complete growth in the Petri dishes, indicating little inhibition of fungal growth; therefore, a direct comparison with the control was not possible, results are presented in Table 4.

(16) $$\%\;inhibition=\frac{\left(Control\;area-Treated\;area\right)}{Control\;area}\times 100$$

*Aspergillus niger*, slight inhibition was observed during the first 48 h with 26.63% and 20.12% inhibition at concentrations of 1 and 3 mg/mL, respectively as shown in Table 5. However, after 120 h, growth stabilized, and inhibition decreased, reaching values below 10%, indicating that the fungus adapted to the medium conditions.

*Penicillium*, on the other hand, showed greater sensitivity to the nanoparticles, especially at 3 mg/mL, where 25.46% inhibition was recorded after 48 h. Although growth increased over time, a slight reduction was maintained compared to the control, with inhibition values close to 11% after 120 h.

Despite a decreasing trend in fungal inhibition over time, this decrease was not statistically significant. Therefore, it is recommended to evaluate higher concentrations of nanoparticles to achieve favorable results for coating applications. Shahidah et al. reported in their research that an increase in TiO_2_ nanoparticle concentration corresponds to greater fungal growth inhibition [59]. Likewise, another study reported that a concentration of Al_2_O_3_ nanoparticles greater than 8 mg/mL showed a greater inhibitory effect than that observed with a concentration of 4 mg/mL [60].

The TiO_2_ nanoparticles can interact with filamentous fungi, but the antifungal efficacy could be limited by the fungal structure defenses involving the oxidative stress mechanism [61]. This capability is related to the interaction between nanoparticles and cell walls, damaging cellular components such as DNA, proteins, and membrane lipids. These nanoparticles are well known as established photocatalysts after light activation, generating reactive oxygen species (ROS) such as hydroxyl radicals and superoxide anions. These are responsible for oxidatively damaged fungal cell wall components such as chitin and β-glucans, disrupting over the cytoplasmic membrane inhibiting spore generation and hyphal extension [62,63].

Based on ROS generation, the biological efficacy of nanoparticles is established by electrostatic interactions of particle adhesion. Preočanin et al. reported that the surface TiO_2_ nanoparticles charge depends on the pH, characterized by a point of zero charge (PZC) typically ranging from 5.8 to 6.2 [64]. TiO_2_ surfaces become predominantly positively charged in acidic environments, creating an electrostatic attraction toward fungal cell walls that have a negative charge due to exposed phosphate and carboxylate groups. This interaction is relevant for *Aspergillus niger* and *Penicillium* spores, maintaining a negative zeta potential under acidic conditions owing to their melanin-rich outer layer [65,66,67].

The differences observed between both fungal structures likely stem from these biological responses. The dense melanin layer in *Aspergillus niger* spores provides a robust shield against oxidative attack. In contrast, the generally thinner cell walls and lower pigment levels found in *Penicillium* species offer less protection, which may explain their slightly higher sensitivity to the treatment [60].

## 4. Conclusions

The main objective of this study was to develop and evaluate a green synthesis route for TiO_2_ nanoparticles using *Cymbopogon citratus* extract and to assess their effectiveness as functional additives in water-based acrylic coatings for improving photocatalytic, hydrophobic, and antifungal properties on construction surfaces. Titanium dioxide nanoparticles synthesized using green chemistry with *Cymbopogon citratus* extract demonstrated remarkable structural and functional properties for application in self-cleaning and antifungal coatings. XRD, SEM, and EDS characterization confirmed nanoparticles with spherical and irregular morphology and an anatase-phase crystalline structure, with an average size of 13.25 nm. In terms of self-cleaning, samples containing 5 wt% TiO_2_ achieved 96.4% methylene blue degradation after 24 h of irradiation, demonstrating excellent photocatalytic performance. Furthermore, the contact angle increased to 104.25°, increasing hydrophobicity by 30.9% compared to the control and enhancing surface self-cleaning. Moreover, nanoparticles showed moderate inhibition against *Penicillium* and *Aspergillus niger*, with greater effectiveness at concentrations of 3 mg/mL, suggesting their potential use as an antifungal additive in coatings. However, it is recommended to evaluate them at higher concentrations and optimize exposure conditions to maximize their efficacy. Overall, the results confirm that titanium dioxide nanoparticles obtained through green synthesis represent a sustainable and efficient alternative for the development of multifunctional coatings, combining photocatalytic, hydrophobic, and antifungal properties with potential applications in construction materials and even the textile industry.

## Figures and Tables

**Table 1:** Average b* coordinate values and methylene blue degradation efficiency for the control sample and TiO_2_ nanoparticle.

Control					
Sample	b* av. (0 h)	b* av. (4 h)	b* av. (24 h)	*AM* (4 h) % av.	*AM* (24 h) % av.
5 mg/L	−21.43	−14.87	−10.81	30.34	49.26
10 mg/L	−14.94	−8.45	−4.65	43.71	69.29
20 mg/L	−9.91	−4.11	−2.80	46.98	76.93
TiO_2_ 1% *w*/*w*					
5 mg/L	−9.96	−2.81	−1.09	71.87	89.08
10 mg/L	−17.15	−8.23	−4.97	51.70	71.13
20 mg/L	−25.51	−15.78	−10.95	38.17	57.09
TiO_2_ 5% *w*/*w*					
5 mg/L	−8.74	−0.59	−0.31	93.31	96.39
10 mg/L	−17.54	−5.76	−2.55	67.23	85.46
20 mg/L	−25.26	−12.65	−8.13	49.88	67.69

av. = Average.

**Table 2:** Summary of contact angle measurements in modified paints.

Modified Coating	Statistical Measure	Left Contact Angle (°)	Right Contact Angle (°)	Average Contact Angle (°)
0% *w*/*w* (control)	mean	84.28	85.32	84.80
	standard deviation	3.24	3.85	3.54
	min	80.56	81.09	80.83
	max	86.52	88.65	87.59
1% *w*/*w* TiO_2_	mean	91.61	92.45	92.03
	standard deviation	0.48	1.51	0.99
	min	91.11	91.27	91.19
	max	92.07	94.15	93.11
5% *w*/*w* TiO_2_	mean	104.83	103.63	104.24
	standard deviation	4.87	5.47	5.17
	min	100.27	100.30	100.28
	max	109.96	109.96	109.96

**Table 3:** Values corresponding to the surface energy of each coating.

Modified Coating	Value
0% *w*/*w* (control)	79.45
1% *w*/*w* TiO_2_	70.28
5% *w*/*w* TiO_2_	54.88

**Table 4:** Average growth area results (mm^2^) of *Aspergillus niger* and *Penicillium*.

Tested Microorganism	Incubation Time	Control (mm^2^)	TiO_2_ (mm^2^)	
			1 mg/mL	3 mg/mL
*Aspergillus niger*	48 h	214.33	157.26	171.20
	120 h	1866.73	1729.93	1681.70
*Penicillium*	48 h	51.49	46.18	38.38
	120 h	716.64	660.55	637.82

**Table 5:** Inhibition (%) results of *Aspergillus niger* and *Penicillium* fungi.

Tested Microorganism	Incubation Time	TiO_2_	
		1 mg/mL	3 mg/mL
*Aspergillus niger*	48 h	26.63	20.12
	120 h	7.33	9.91
*Penicillium*	48 h	10.31	25.46
	120 h	7.83	11.00

## Data Availability

The original contributions presented in this study are included in the article. Further inquiries can be directed to the corresponding author.

## References

[1] Asha A.B., Narain R. (2020). Nanomaterials properties. Polymer Science and Nanotechnology.

[2] El Nady J., Kashyout A.B., Ebrahim S., Soliman M.B. (2016). Nanoparticles Ni electroplating and black paint for solar collector applications. Alex. Eng. J..

[3] Ahmad I., Kan C. (2017). Visible-Light-Driven, Dye-Sensitized TiO_2_ Photo-Catalyst for Self-Cleaning Cotton Fabrics. Coatings.

[4] Nosrati R., Olad A., Shakoori S. (2017). Preparation of an antibacterial, hydrophilic and photocatalytically active polyacrylic coating using TiO_2_ nanoparticles sensitized by graphene oxide. Mater. Sci. Eng. C.

[5] Sardella D., Gatt R., Valdramidis V.P. (2017). Physiological effects and mode of action of ZnO nanoparticles against postharvest fungal contaminants. Food Res. Int..

[6] Wang C., Guo J., Yu H., Lei H., Wang Z., Zhao M., Li J., Li X. (2021). Preparation and self-cleaning property of a superhydrophobic coating based on micro–nano integrated TiO_2_ microspheres. Ceram. Int..

[7] Solano R., Patiño-Ruiz D., Herrera A. (2020). Preparation of modified paints with nano-structured additives and its potential applications. Nanomater. Nanotechnol..

[8] Chen M.C., Koh P.W., Ponnusamy V.K., Lee S.L. (2022). Titanium Dioxide and Other Nanomaterials-Based Antimicrobial Additives in Functional Paints and Coatings: A Review. Prog. Org. Coat..

[9] Chen J., Gao J., Liu X., Wang P., Yu X., Zhao F., Sun Y., Feng W., Wang Q. (2022). Controllable Phase Transformation and Enhanced Photocatalytic Performance of Nano-TiO_2_ by Using Oxalic Acid. Nanomaterials.

[10] Poppi G., Colombini E., Salvatori D., Balestri A., Baldi G., Leonelli C., Veronesi P. (2022). A Multi-Physic Modelling Insight into the Differences between Microwave and Conventional Heating for the Synthesis of TiO_2_ Nanoparticles. Processes.

[11] Herrera-Rivera R., Morales-Bautista J., Pomar F.S., Quijano-Briones J.J., Eguía-Eguía S., Olvera M.D.L.L., Martínez-Huerta A., Pérez-Tijerina E. (2025). Synthesis of TiO_2_ Nanoparticles by Precipitation Method and Its Photocatalytic Activity: Experimental Design by Taguchi Method. J. Nanotechnol..

[12] Andronic L., Ghica D., Stefan M., Mihalcea C.G., Vlaicu A.-M., Karazhanov S. (2022). Visible-Light-Active Black TiO_2_ Nanoparticles with Efficient Photocatalytic Performance for Degradation of Pharmaceuticals. Nanomaterials.

[13] Salehzadeh J., Nassiri M., Dehghani Z., Mohajeri S. (2025). Synthesis of TiO_2_ nanoparticles: Structural characterization, Photocatalytic Degradation of Malachite Green Dye from Aqueous Solution under UV Irradiation. J. Indian Chem. Soc..

[14] Deliza S.L.R., Liandi A.R., Putri R.A., Safni S. (2025). Green Synthesis Approach on Fabrication of TiO_2_ Nanoparticle Using Peel Extract of *Baccaurea racemosa* for Photocatalytic Degradation of Acid Red-185. Environ. Nanotechnol. Monit. Manag..

[15] Iravani S. (2011). Green synthesis of metal nanoparticles using plants. Green Chem..

[16] Ahmed S., Ahmad M., Swami B.L., Ikram S. (2016). A review on plants extract mediated synthesis of silver nanoparticles for antimicrobial applications: A green expertise. J. Adv. Res..

[17] Patiño-Ruiz D., Sánchez-Botero L., Tejeda-Benitez L., Hinestroza J., Herrera A. (2020). Green Synthesis of Iron Oxide Nanoparticles Using Cymbopogon citratus Extract and Sodium Carbonate Salt: Nanotoxicological Considerations for Potential Environmental Applications. Environ. Nanotechnol. Monit. Manag..

[18] Uthiravel V., Narayanamurthi K., Raja V., Anandhabasker S., Kuppusamy K. (2024). Green Synthesis and Characterization of TiO_2_ and Ag-Doped TiO_2_ Nanoparticles for Photocatalytic and Antimicrobial Applications. Inorg. Chem. Commun..

[19] Ghareeb A., Fouda A., Kishk R.M., El Kazzaz W.M. (2024). Unlocking the Potential of Titanium Dioxide Nanoparticles: An Insight into Green Synthesis, Optimizations, Characterizations, and Multifunctional Applications. Microb. Cell Fact..

[20] Sangeetha A., Ambli A., Nagabhushana B.M. (2024). Green and Chemical Synthesis of TiO_2_ Nanoparticles: An In-Depth Comparative Analysis and Photoluminescence Study. Nano Struct. Nano Objects.

[21] Anandgaonker P., Kulkarni G., Gaikwad S., Rajbhoj A. (2019). Synthesis of TiO_2_ nanoparticles by electrochemical method and their antibacterial application. Arab. J. Chem..

[22] Praveen P., Viruthagiri G., Mugundan S., Shanmugam N. (2014). Structural, optical and morphological analyses of pristine titanium di-oxide nanoparticles—Synthesized via sol-gel route. Spectrochim. Acta Part A Mol. Biomol. Spectrosc..

[23] Bellotti N., Romagnoli R., Quintero C., Domínguez-Wong C., Ruiz F., Deyá C. (2015). Nanoparticles as antifungal additives for indoor water borne paints. Prog. Org. Coat..

[24] Beeldens A., Leuven K.U. (2007). Photocatalysis of Cementitious Materials. https://www.researchgate.net/publication/282916054.

[25] Guo M.Z., Maury-Ramirez A., Poon C.S. (2016). Self-cleaning ability of titanium dioxide clear paint coated architectural mortar and its potential in field application. J. Clean. Prod..

[26] Gharbi A.H., Laouini S.E., Hemmami H., Bouafia A., Gherbi M.T., Ben Amor I., Hasan G.G., Abdullah M.M., Trzepieciński T., Abdullah J.A.A. (2024). Eco-Friendly Synthesis of Al_2_O_3_ Nanoparticles: Comprehensive Characterization Properties, Mechanics, and Photocatalytic Dye Adsorption Study. Coatings.

[27] Moreno A.D., Schuster J.M., Manzur J., Rosenberger M.R. Modeling of the Evaporation of a Sessile Droplet on a Solid Surfacea 2015. https://www.researchgate.net/publication/304215062.

[28] Stalder A.F., Kulik G., Sage D., Barbieri L., Hoffmann P. (2006). A snake-based approach to accurate determination of both contact points and contact angles. Colloids Surf. A Physicochem. Eng. Asp..

[29] Das K., Tiwari R.K.S., Shrivastava D.K. (2010). Techniques for evaluation of medicinal plant products as antimicrobial agent: Current methods and future trends. J. Med. Plants Res..

[30] Moreno-Vargas J.M., Echeverry-Cardona L.M., Moreno-Montoya L.E., Restrepo-Parra E. (2023). Evaluation of Antifungal Activity of Ag Nanoparticles Synthetized by Green Chemistry against Fusarium solani and Rhizopus stolonifera. Nanomaterials.

[31] Sahraoui A., Hamlaoui M., Chikhi S., Harrat S., Baghriche O., Zertal A., Vernuccio S. (2025). Green Synthesis and Characterization of Titanium Dioxide Nanoparticles Using Eucalyptus globulus Leaf Extract: Impacts of the Mild Thermal Treatment. Mater. Today Sustain..

[32] Rabe A.M., Aliero B.L., Galadima A., Baqi A.S. (2018). Thermal Decomposition of Camel Grass and Lemon Grass. J. Sci. Res. Rep..

[33] Fabiyi O.A., Olatunji G.A., Adebayo M.O., Atolani O. (2018). Effect of Thermal Degraded Products of *Cymbopogon citratus* on the In Vitro Survival of *Meloidogyne incognita* Eggs and Juveniles. Ceylon J. Sci..

[34] Solano R.A., Herrera A.P., Maestre D., Cremades A. (2019). Fe–TiO_2_ Nanoparticles Synthesized by Green Chemistry for Potential Application in Wastewater Photocatalytic Treatment. J. Nanotechnol..

[35] Parvez M.S., Rahman A., Habib A.K.M.A., Rokon S.M.N. (2024). Green Synthesis of Undoped and Yttrium, Bismuth Co-Doped Titanium Dioxide Nanoparticles Using *Bryophyllum pinnatum* for Photocatalytic Application. Results Mater..

[36] Korkmaz N., Ceylan Y., Kısa D., Güçlü E., Şen F., Karadağ A. (2025). Environmentally Friendly Synthesis and Biological Activity Assessment of Titanium Dioxide Nanoparticles with Hemp (*Cannabis sativa* L.) Leaf Extract. Next Mater..

[37] Moeini-Eghbali N., Eshghi H. (2024). Immobilized Nickel Nanoparticles on Modified Magnetic Titanium Dioxide: A Proficient and Eco-Friendly Nanocatalyst for the Green A^3^-Coupling Synthesis of Propargylamines. J. Mol. Struct..

[38] Parasuraman V., Sekar P.P., Lee H., Sheraz M., Ly H.N., Azizar G.A.B., Hong J.W., Lee W.R., Kim S. (2024). Photocatalytic Self-Cleaning Eco-Friendly Paint: A Unique Approach for Efficient Indoor Air Pollutant Removal and Surface Disinfection. Constr. Build. Mater..

[39] Padmanabhan N.T., John H. (2020). Titanium Dioxide Based Self-Cleaning Smart Surfaces: A Short Review. J. Environ. Chem. Eng..

[40] Liu Z., Wang R., Kan F., Jiang F. (2014). Synthesis and characterization of TiO_2_ Nanoparticles. Asian J. Chem..

[41] Sangeetha M., Kalpana S., Senthilkumar N., Senthil T.S. (2024). Investigation on Visible-Light Induced Photocatalytic Activity for Pure, Ce:Doped TiO_2_ and B:Ce Co-Doped TiO_2_ Catalysts. Optik.

[42] Khan S., Noor T., Iqbal N., Yaqoob L. (2024). Photocatalytic Dye Degradation from Textile Wastewater: A Review. ACS Omega.

[43] Fujishima A., Zhang X., Tryk D.A. (2008). TiO_2_ photocatalysis and related surface phenomena. Surf. Sci. Rep..

[44] Chen J., Poon C.S. (2009). Photocatalytic construction and building materials: From fundamentals to applications. Build. Environ..

[45] Alonso E., Montequi I., Cocero M.J. (2009). Effect of synthesis conditions on photocatalytic activity of TiO_2_ powders synthesized in supercritical CO_2_. J. Supercrit. Fluids.

[46] Aghababaei E., Alizadeh M., Bahrami A. (2025). Synthesis and Characterization of Ce-Doped TiO_2_/Cu-Doped Fe_3_O_4_ Heterogeneous Photocatalyst for Antibacterial Applications and Visible-Light Photocatalytic Degradation of Methylene Blue. Ceram. Int..

[47] Handayani N., Rinovian A., Habibillah M.R., Rozana M., Wellia D.V., Anshori I., Zulfikar M.A., Nasir M. (2025). Electrospun Recycled Nylon/Titanium Dioxide Nanofiber Composite for Photocatalytic Degradation of Methylene Blue. J. Sci. Adv. Mater. Devices.

[48] Islam M.T., Dominguez A., Turley R.S., Kim H., Sultana K.A., Shuvo M.A.I., Alvarado-Tenorio B., Montes M.O., Lin Y., Gardea-Torresdey J. (2020). Development of Photocatalytic Paint Based on TiO_2_ and Photopolymer Resin for the Degradation of Organic Pollutants in Water. Sci. Total Environ..

[49] Wu H., Lu X., Feng Z., He R. (2025). Dynamic Hydrophobicity and Surface Reconstruction Mechanism of Self-Cleaning Traffic Marking Coatings Incorporating Modified TiO_2_ Nanoparticles. Constr. Build. Mater..

[50] Saba M., Coronado-Hernández O.E., Gil L.K.T. (2024). Energy Efficiency in Subtropical Homes: Replacing Asbestos–Cement Roofs with Sustainable Alternatives. Buildings.

[51] Saba M., Torres Gil L.K., Chanchí Golondrino G.E. (2023). Physicochemical Analysis of Primers and Liquid Membranes as Asbestos’ Encapsulant. Constr. Build. Mater..

[52] Gouda A.H., Donia N.S., Khalil M.M.H., El-Gendy A.S. (2024). Development of epoxy coating with TiO_2_ nanoparticles for self-cleaning applications. Innov. Infrastruct. Solut..

[53] Kocijan A., Conradi M., Hočevar M. (2019). The Influence of Surface Wettability and Topography on the Bioactivity of TiO_2_/Epoxy Coatings on AISI 316L Stainless Steel. Materials.

[54] Takwa L.V. (2021). Spin Coating and Characterization of Self-Cleaning TiO_2_ Thin Films for Photovoltaic Application. Master’s Thesis.

[55] Sajadinia Z., Arabian D., Charmi M. (2025). Production of a Green Organic Multifunctional Anticorrosion Coating Composed of Stearic Acid and Nanoparticles for Concrete Using a Simple and Cost-Effective Method. Prog. Org. Coat..

[56] Huang J.-Y., Lai Y.-K., Aliofkhazraei M. (2015). TiO_2_-Based Surfaces with Special Wettability—From Nature to Biomimetic Application. Wetting and Wettability.

[57] Liu Z., Zhou K., Wang J., Peng C., Li Y., Yang S., Tao E. (2025). Modulation of Titanium Dioxide/Graphene Interface by Mg^2+^ Doping: Titanium Dioxide Coatings with Conductive, Corrosion-Resistant and Superhydrophobic Features. Energy.

[58] Yang R., Zhao X., Li Y., Wang J., Zhou K., Yang S., Li Y. (2025). Feδ^+^ Diaspora Titanium Dioxide and Graphene: A Study of Conductive Powder Materials and Coating Applications. J. Colloid Interface Sci..

[59] Ahmad N.S., Abdullah N., Yasin F.M. (2019). Antifungal Activity of Titanium Dioxide Nanoparticles against Candida albicans. BioResources.

[60] Manyasree D., Kiranmayi P., Kumar R. (2018). Synthesis, Characterization and Antibacterial Activity of Aluminium Oxide Nanoparticles. Int. J. Pharm. Pharm. Sci..

[61] Ghareeb A., Fouda A., Kishk R.M., El Kazzaz W.M. (2025). Multifaceted Biomedical Applications of Biogenic Titanium Dioxide Nanoparticles Fabricated by Marine Actinobacterium Streptomyces vinaceusdrappus AMG31. Sci. Rep..

[62] Foster H.A., Ditta I.B., Varghese S., Steele A. (2011). Photocatalytic Disinfection Using Titanium Dioxide: Spectrum and Mechanism of Antimicrobial Activity. Appl. Microbiol. Biotechnol..

[63] Slavin Y.N., Bach H. (2022). Mechanisms of Antifungal Properties of Metal Nanoparticles. Nanomaterials.

[64] Preočanin T., Kallay N. (2006). Point of zero charge and surface charge density of TiO_2_ in aqueous electrolyte solution as obtained by potentiometric mass titration. Croat. Chem. Acta.

[65] Wargenau A., Fleissner A., Bolten C.J., Rohde M., Kampen I., Kwade A. (2011). On the Origin of the Electrostatic Surface Potential of *Aspergillus niger* Spores in Acidic Environments. Res. Microbiol..

[66] Mahnič N., Pavlovič B., Vidrih R., Bohinc K., Štukelj R., Kovač M., Jeršek B. (2025). *Penicillium expansum* Adhesion to Materials Used in Food Industry. J. Adhes..

[67] Fernandes S., Simões L.C., Lima N., Simões M. (2019). Adhesion of Filamentous Fungi Isolated from Drinking Water under Different Process Conditions. Water Res..

